# Artificial Intelligence and Big Data in Public Health

**DOI:** 10.3390/ijerph15122796

**Published:** 2018-12-10

**Authors:** Kurt Benke, Geza Benke

**Affiliations:** 1 School of Engineering, University of Melbourne, Parkville, Victoria 3010, Australia; 2 AgriBio, Centre for AgriBiosciences, State Government of Victoria, Bundoora, Victoria 3083, Australia; 3 School of Public Health and Preventive Medicine, Monash University, 553 St Kilda Rd, Melbourne 3004, Australia; geza.benke@monash.edu

**Keywords:** algorithms, Big Data, machine learning, deep learning, data mining, visualization, epidemiology, predictive analytics, precision medicine, vision, wearable AI

## Abstract

Artificial intelligence and automation are topics dominating global discussions on the future of professional employment, societal change, and economic performance. In this paper, we describe fundamental concepts underlying AI and Big Data and their significance to public health. We highlight issues involved and describe the potential impacts and challenges to medical professionals and diagnosticians. The possible benefits of advanced data analytics and machine learning are described in the context of recently reported research. Problems are identified and discussed with respect to ethical issues and the future roles of professionals and specialists in the age of artificial intelligence.

## 1. Introduction

Big Data is associated with the massive computational resources needed to cope with the increasing volume and complexity of data from many sources, such as the internet and remote sensor networks. Big Data includes information that is structured, semi-structured, or unstructured, and there may be complex interrelationships that are syntactic, semantic, social, cultural, economic, and organizational in nature. There may also be epistemic uncertainties that enter the data processing pipeline and hinder decision making by humans and computers, including (a) data corruption by noise and artefacts, (b) data entry errors, (c) duplicate records, (d) missing records, and (e) incomplete digital records relating to information on date, age, gender, or other variables.

The Big Data culture embraces cyber-physical systems, cloud computing, and the Internet of Things (IoT), also known as Industry 4.0. These massive data processing systems often involve significant levels of process automation. The current global focus on Big Data on the internet has been coupled with the rise of artificial intelligence (AI) in diagnostics and decision making following recent advances in computer technology. An early catalyst was the rapid growth of social media and the rapidly declining cost of microelectronics in connected devices.

In this paper, the fundamentals of AI and Big Data are introduced and described in the context of public health. Examples of potential benefits and critical comments are provided within the framework of recent research published in many prestigious journals such as *JAMA* and *Nature Reviews* [1,2,3,4,5,6,7,8,9,10,11]. The examples provided are illustrative but not exhaustive, and were chosen to cover a variety of interests in public health, such as epidemiology, precision medicine, medical screening, vision augmentation, and psychology. Questions are raised on ethical issues and the implications for the future roles of specialists and expert opinion. Possible answers are discussed and evaluated, and we argue the need to start the conversation on engagement with AI by professionals in the public health sector. 

## 2. Medical Research 

Big Data is often associated with large-scale macrosystems with distributed data processing that is often beyond the capability of local desktop computers and traditional software because of constraints imposed by speed and volume of processing. Information processing is diverse and may include the streaming of text messages, images, video, and music files. The following five characteristics of Big Data have been quoted in the past and can occur in different combinations (see also [1,12,13]):

- Variability (lack of structure, consistency, and context);
- Variety (includes audio files, imagery, numerical data, and text data);
- Velocity (real-time processing and very high speed of transmission);
- Veracity (accuracy, noise, and uncertainty in data);
- Volume (extremely large data sets).

The quantity and quality of Big Data are influenced by statistical replications in large-scale scientific experiments and are subject to many epistemic uncertainties. The most significant social impacts of Big Data are likely to occur when it is used in combination with artificial intelligence.

In epidemiology, the huge potential of Big Data was illustrated by a pioneering study reported by Deiner [1,13], who demonstrated that the spread of epidemics can be detected early by tracking online queries on disease symptoms using social media such as Google Search and Twitter. Pattern recognition and data analytics were used as tools for the detection, recognition, and classification of patterns of disease relating to the incidence of conjunctivitis. The study results suggested that early warning and detection of biosecurity threats and epidemics of influenza may be possible by the surveillance of online queries. Timeliness of detection and early intervention are obvious benefits subject to further validation by correlation with hard data, including electronic medical records, medication sales, and hospital admissions. 

The accuracy of data from social media is affected by limited online access by minority groups and participation by age, gender, or ethnicity. Aside from Google search errors, Twitter queries may be compromised by ambiguities involving Boolean logic using If–Then–Else rules, resulting in incorrect predictions for disease incidence.

Such internet-based approaches have uncertainties that will need to be addressed in future research, such as the accuracy of search algorithms, updating rates, and the authenticity of data [1]. A “hot topic” in the mass media is the revelation that some governments and some data analytics companies have sought to influence public opinion by spreading so-called “fake news” and “fake” Twitter accounts. This problem may be addressed by the application of AI-based screening methods to check online Big Data. For example, a machine learning algorithm such as a neural network could be trained to detect certain recurring words, phrases, and views expressed in social media and then use pattern recognition to correlate the data with news content, candidate names, media outlets, and geographic locations. Suspicious correlations and clusters can be revealed in this manner.

Another example of a Big Data application is precision medicine, where clinical trials are based on patient selection according to DNA profiling, which provides biomarkers for targeted treatment, rather than a standard approach used for the whole population [2]. The selection of individuals sharing the same genetic abnormality for clinical trials leads to more precise drug development and more accurate treatments with existing drugs [2,3]. Additional advantages include reductions in sample size and reduced variability in clinical trials—without the need for a placebo. There is also the potential for genome sequencing to produce greater diagnostic sensitivity and more precise treatment [4]. Large-scale population studies and Big Data analytics can be used for more accurate genotypic and phenotypic data to support investigations of causality.

There are many uncertainties in the precision medicine approach, including whether genetic profiling is more effective than other factors, such as changes in lifestyle and exposure to environmental stressors [14]. Response to drug treatment is affected by diet, exercise, medication, alcohol, tobacco, and the microbiome in the digestive tract [5]. Information on these factors needs to be provided within a comprehensive Big Data framework. Large-scale population studies are required for information discovery to resolve many of these uncertainties.

The development of expensive genetically-tailored medicines may benefit only a small minority of wealthy citizens. Would limited funding be better deployed on medical research that benefits the majority? The genetic basis of a disease in some cases is secondary in importance to environmental factors, and behavior modification relating to diet or exercise may be just as effective but less expensive. In addition, an expensive new drug developed from a clinical trial may be no more effective than a combination of existing inexpensive drugs. In an era of declining research funding, there is a case for data analytics and the investigation of costs vs. benefits and trade-offs associated with all areas of research. 

Important challenges relating to the development of ethics for precision medicine include rights to ownership of genetic information, privacy, control of its dissemination, and potential consumer use or abuse. For example, unauthorized knowledge of genomic information can influence consumer risk perception in personnel selection processes or credit ratings. The DNA of an individual is not a password that can be changed. There is a need for greater debate on genetic privacy, such as finding the right balance between public rights to privacy and evidence required for law enforcement, and potential improvements in regulatory control [11,15].

## 3. Artificial Intelligence

Artificial intelligence is a generic term for a machine (or process) that responds to environmental stimuli (or new data) and then modifies its operation to maximize a performance index [16,17]. In practice, the learning process is implemented using mathematics, statistics, logic, and computer programming. The learning process enables the AI model to be trained on data in an iterative procedure with parameter adjustment by trial and error using reinforcement rules. The performance index may be the minimization of the difference between model predictions and experimental data.

**Figure 1:**
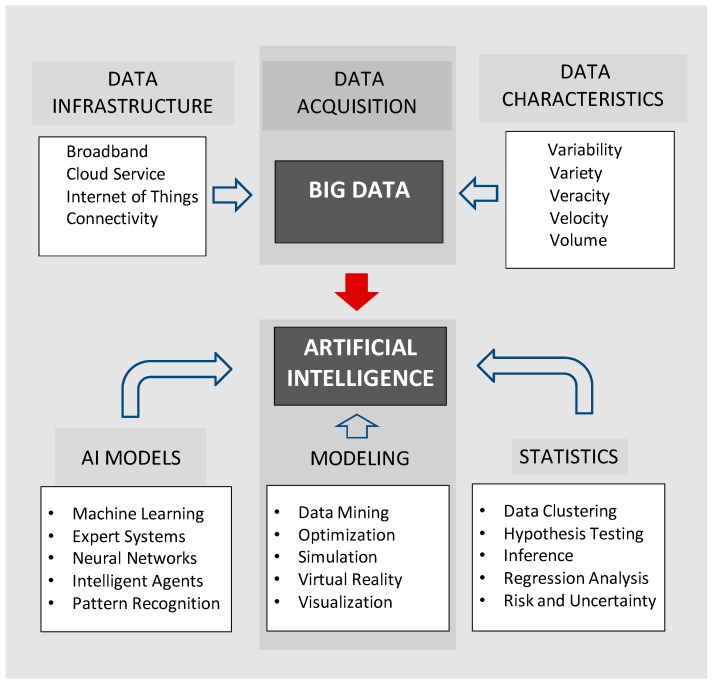
Artificial intelligence (AI) and Big Data.

The training phase is based on the classical scientific method, which has origins in antiquity with Aristotle and was formalized in the writings of Francis Bacon (circa. 1600). Once trained, the AI model can be applied to new data to make decisions. Decision making can involve detection, discrimination, and classification. This approach is known as supervised learning because it is analogous to an adaptive system with a feedback loop and a goal-directed learning process. Examples include the diagnosis of a disease from pathology results, or assigning the correct names to labeled images of different skin cancers. The potential role of AI in processing Big Data in precision medicine has been suggested by experts due to the massive resources needed for predictive analytics [18]. The potential of Big Data is most apparent when viewed as a database for artificial intelligence (Figure 1).

Some AI models carry out unsupervised learning by a clustering process, such as the *k*-means clustering algorithm, with the aim of discovering important groupings or defining features in the data [17]. A major challenge in the future development of AI is how to exploit Big Data to produce high-level abstractions that can simulate a human subjective response. In a clinical context, there is potential for the development of a digital expert that facilitates automatic conversion of pathology results into a written report or a verbal explanation.

Examples of AI approaches include machine learning, pattern recognition, expert systems, and fuzzy logic. Many AI models have strong statistical underpinnings and have learning rules based on nonlinear optimization procedures. The typical artificial neural network (ANN) consists of a predefined architecture with a matrix of weights that are adjusted iteratively according to the defined performance index [6,17].

## 4. Machine Learning

Machine learning is a subfield of AI that is based on training on new data using an adaptive approach, such as a neural network, but without explicit programming of new rules as required by other types of AI algorithms such as expert systems [16,17]. The coefficients (weights) of the model are adjusted iteratively, with repeated presentations of the data, until the performance index is optimized. The recent investigation by Gulshan et al. [6] reported the validation of a “deep learning” algorithm for the detection of diabetic retinopathy and diabetic macular edema (DME). The study produced high sensitivity (87%) and specificity (98%), which exceeded the performance of human specialists. The deep learning approach did not use explicit feature measurements, such as lines or edges in images, as in traditional pattern recognition. It was instead trained on a very large set of labeled images, and discrimination was based on using the entire image as a pattern (i.e., without explicit feature extraction), followed by validation on test images from a database. Very large training sets can help to compensate for image variability without the need for canonical transformations to normalize for various geometrical aberrations.

The deep learning algorithm consisted of a convolutional neural network with a very large number of hidden layers optimized using a multistart gradient descent procedure [19]. Very high processing speeds were achieved in the learning phase using graphics cards and parallel processing with thousands of images. The classification results were very impressive and have been echoed in similar studies reviewed elsewhere, raising questions about the future roles of human specialists in medical diagnostics [7,20].

The results were deemed to be so significant that *JAMA* published two invited editorials together with a viewpoint article as commentaries on the machine learning procedure as a digital diagnostician [7,8,9]. Future research is still needed on performance in clinical settings and improvements in the labeling scheme used for training data, which was based on a majority vote from medical experts. The performance index could be improved by the use of more objective metrics for detection of DME, such as optical coherence tomography.

Jha and Topol [9] suggested that adaptation to the challenge of machine learning by medical specialists may be possible if they become generalists and information professionals. Humans could use AI-based automated image classification for screening and detection, but would manage and integrate the information in a clinical context, advise on additional testing, and provide guidance to clinicians. However, this potential symbiosis assumes that AI is restricted to pattern recognition and image classification using machine learning algorithms.

In practice, different AI models can also include systems that can apply logic, inference, strategy, and data integration. This is demonstrated by software such as IBM’s “Deep Blue” and “Watson” and Google’s “DeepMind”, which have employed AI algorithms to defeat the best human experts at strategy games such as chess and GO and win the televised *Jeopardy* general knowledge quiz by accessing the entire knowledge base of Wikipedia. Together with software personal assistants such as Apple’s “Siri” and Microsoft’s “Cortana” (which provide conversational response and advice), there is a sense that specialized AI algorithms are evolving rapidly in sophistication. Recently, internet giants Amazon (with “Alexa”) and Google (with “Assistant”) have been reported to be seeking business partners for further development of the next generation of personal assistants, which will feature voice-activated platforms for interactive phone calls that imitate humans.

Human infants learn by a process of trial and error and acquiring a series of specialist skills by interaction and eventual combination (e.g., crawling, walking, and talking). In a similar manner, cooperation between specialist AI algorithms (including machine learning) could one day simulate the human cognitive process to produce generalized AI. For example, a suite of specialized programs could be controlled in a top-down manner by a master AI program with a standard set of rules and axioms (analogous to encoded DNA). Several companies such as Arria NLG, Retresco, and others have already demonstrated that AI and automatic language processing can convert diagnostic data into a final report and verbal counseling to replace a human expert. 

The qualitative distinction between AI and human intelligence is arguably a matter of degree only, as measured by the famous Turing test. In the long term, AI may converge with and even surpass human judgment, given current rapid developments in software and hardware coupled with feedback loops incorporating multispectral IoT sensors and complexity theory. Progress in AI applications in recent years has been exponential, not linear, and is now ubiquitous and immersive in our lifestyles. 

There is clearly a need for widespread discussion and debate on the future roles of human experts, diagnosticians, and medical specialists. The development of ethical standards is necessary for the application of AI in the medical sector to support regulations for the protection of human rights, user safety, and algorithmic transparency. Algorithms need to be free of bias in coding and data and should be explanatory if possible and not just predictive. Controlling the pace of automation would ensure incremental adoption, if necessary, with constant process evaluation [18].

## 5. Vision Augmentation

The OrCam portable device for artificial vision employs a miniature television camera mounted on the frame of spectacles for optical character recognition by a microchip [10,21]. Both text and patterns, including faces, can be converted by an algorithm into words heard through an earpiece. This is an example of an AI-based wearable device [22]. A recent evaluation of the OrCam for blind patients produced very encouraging results [10].

Device evaluation was based on a 10-item test that included reading a menu, reading a distant sign, and reading a headline in a newspaper. The score of the evaluation was the number of test items correctly vocalized. Because the items were not weighted by importance, the development of a weighting scheme would further facilitate comparison with similar devices, such as NuEyes [23]. A conventional eye chart could be used as a baseline.

The potential of such a noninvasive AI device for sensory substitution cannot be overemphasized. Even texture and color could be digitized and identified as words through the earpiece. In combination with GPS data and bionic vision [24], a blind person could acquire situational awareness and vision for navigation in outdoor locations. The performance of the OrCam device could be improved by adding text to city pavements with location information such as “road”, “crossing”, or “bus stop”. Text information could be rendered in low contrast to conform with environmental aesthetics. Adding text to walls and floors inside buildings would also enable navigation in new environments while providing continuous information, commentary, and environmental awareness.

## 6. Data Mining

In data science, passive data sets in stored archives are not very useful unless the information can be processed for decision making, forecasting, and data analytics. Data mining is a process for knowledge discovery that is used to find trends, patterns, correlations, anomalies, and features of interest in a database [25,26]. The results can be used for making decisions, making predictions, and conducting quantitative investigations. Data mining has been described as a combination of computer science, statistics, and database management with rapidly increasing utilization of AI techniques (e.g., machine learning) and visualization (with advanced graphics), especially in the case of Big Data analytics [26]. 

Common approaches include multivariate regression analysis and neural networks to construct predictive models. Additional approaches are listed in Figure 1, and many other techniques are described in literature reviews [26,27]. An example of a machine learning application would be to train a neural network to detect novel patterns in patient data and correlations between medications and adverse side effects by scanning electronic medical records for risk assessment [28]. Before data mining it may be necessary to “clean” the stored records with appropriate data cleaning software for the detection and correction of records that may be inaccurate, redundant, incomplete, or corrupt—which are typical epistemic uncertainties [29,30]. Software tools are available from various companies to support data quality and data auditing, including data management products designed for Big Data frameworks (see also Hadoop, Cloudera, MongDB, and Taklend [30,31]). 

Data mining may be enhanced by data visualization techniques that use human perception for information discovery in complex data sets showing no obvious patterns. Visualization enables the exploration and identification of trends, clusters, and artifacts and provides a starting point for detailed numerical analysis [32]. Techniques for visualization include 2D and 3D color graphics; bar graphs; maps; polygons with different shapes, sizes, colors, and textures; video data; and stereo imagery. An example in bioinformatics is the Microbial Genome Viewer, which is a web-based tool used for the interactive graphical combination of genomic data including chromosome wheels and linear genome maps from genome data stored in a MySQL database [33]. 

Basic concepts in data mining and visualization are demonstrated by the following small-scale example in the field of psychology [34]. The correlation between subjective ratings of fear and the appearance and perception of threat were explored in a pilot study involving a mixed-gender group of first-year university students in the age range of 16–43 years (*n* = 31, F = 24, M = 7). The subjects were requested to rank images of 29 animals and insects on a three-point scale (1 = not; 2 = quite; 3 = very) for both appearance and perception of threat. A standard multiple linear regression (MLR) model was fitted to the data in the original study, with fear as the dependent variable, and revealed that the weighting for perception of threat was 0.85 vs. 0.15 for appearance of threat. In the current paper, a standard feed-forward backpropagation artificial neural network (ANN) was trained on the data using commercial software, noting that the very limited statistical data would produce indicative results sufficient for illustration [35]. Both MLR and ANN produced correlation coefficients *r* > 0.90 on training data, thus demonstrating the potential of this approach for predictive analytics. For improved confidence, a future study would need more extensive experiments and greater statistical power using Big Data.

**Figure 2:**
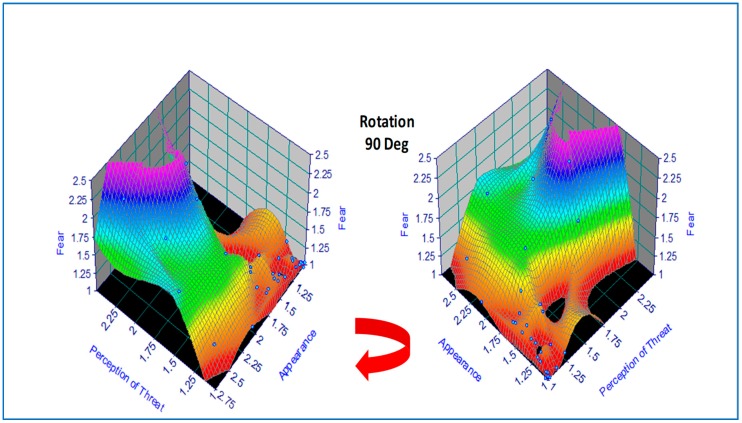
Illustrative example of a response surface for fear as a function of perception of threat and appearance of threat. Visualization reveals discontinuities (two black holes in the right image) exposing regions of zero-fear response under certain combinations of appearance and perception of threat.

For data visualization, the original tabulated data was modeled using a hybrid 2D polynomial function *z* = *f(x,y*) by regression analysis, depicted as a response surface in Figure 2, which was produced using commercial software [36]. An alternative function approximation is possible by ANN but was not used on this occasion due to the small sample size. This would have involved a least-squares error regression analysis with model predictions plotted incrementally along each axis. In the present case, data visualization was accomplished by color coding (where the numerical scale was mapped linearly onto a color scale) followed by a 90 Deg clockwise rotation, revealing two hidden areas of zero-fear response under certain combinations of appearance and perception of threat (e.g., animals that are perceived to be dangerous but are dead or disabled). 

Visualization in color and 3D geometry can therefore increase the probability of information discovery in a database or spreadsheet. The huge volume of information that is characteristic of Big Data suggests that there is potential for greater use in data mining using AI for automation and visualization for initial trend detection. Percepts and gestalts are characteristic of human vision and provide additional tools to augment the operation of data mining.

## 7. Conclusions

The fundamentals of AI and Big Data have been introduced and described in the context of potential applications to data analytics in public health and the medical sciences. The advent of AI and Big Data will change the nature of routine screening in specialist diagnostics and perhaps even in general practice and routine pathology [8,9,10,19,37]. A deeper understanding of these new technologies is required for public health and policy development by bureaucrats, politicians, and business leaders. The important issues of ethics and the need for an overarching regulatory framework for AI and precision medicine have not been sufficiently addressed in public health, and need further attention. These same issues will also have increasing prominence in the public discourse on autonomous cars and battlefield robots.

The combination of AI and Big Data has the potential to have profound effects on our future. The role of the medical specialist will be challenged as these technologies become more widespread and integrated. There is not yet a vigorous debate about the future roles of specialists and diagnosticians if specialist AI programs can perform with greater sensitivity and specificity. Will AI eventually take over for humans in medical diagnostics? Will the future role of the human specialist move to case management, thus leaving screening, detection, and diagnostics to become the domain of intelligent machines?

It has been argued that these questions may be answered within a framework that allows human experts to occupy new roles as generalists and information specialists [9,10]. For example, AI and automation could be used as a front-end for diagnostics involving the interpretation of pathology results and image classification. This would leave humans free to manage and integrate the information in a clinical context, advise on additional testing if necessary, and provide continuing guidance to clinicians and patients. This approach is feasible in the short term and medium term with specialist AI, but will this approach suffice in the longer term with the rise of generalized AI?
